# Phenolic Profile, Antioxidant Capacity and Antimicrobial Activity of Nettle Leaves Extracts Obtained by Advanced Extraction Techniques

**DOI:** 10.3390/molecules26206153

**Published:** 2021-10-12

**Authors:** Ivona Elez Garofulić, Valentina Malin, Maja Repajić, Zoran Zorić, Sandra Pedisić, Meta Sterniša, Sonja Smole Možina, Verica Dragović-Uzelac

**Affiliations:** 1 Faculty of Food Technology and Biotechnology, University of Zagreb, 10000 Zagreb, Croatia; ivona.elez@pbf.unizg.hr (I.E.G.); zoran.zoric@pbf.unizg.hr (Z.Z.); sandra.pedisic@pbf.unizg.hr (S.P.); vdragov@pbf.hr (V.D.-U.); 2 Biotechnical Faculty, University of Ljubljana, 1000 Ljubljana, Slovenia; valentina.malin@bf.uni-lj.si (V.M.); meta.sternisa@bf.uni-lj.si (M.S.); sonja.smole-mozina@bf.uni-lj.si (S.S.M.)

**Keywords:** nettle leaves, microwave-assisted extraction, pressurized liquid extraction, advanced extraction techniques, plant polyphenols, antioxidant capacity, ORAC, UPLC MS^2^, antimicrobial activity

## Abstract

Nettle is a widely known plant whose high biological activity and beneficial medicinal effects are attributed to various bioactive compounds, among which polyphenols play an important role. In order to isolate polyphenols and preserve their properties, advanced extraction techniques have been applied to overcome the drawbacks of conventional ones. Therefore, microwave-assisted extraction (MAE) has been optimized for the isolation of nettle leaves polyphenols and it was compared to pressurized liquid extraction (PLE) and conventional heat-reflux extraction (CE). The obtained extracts were analyzed for their individual phenolic profile by UPLC MS^2^ and for their antioxidant capacity by ORAC assay. MAE proved to be the more specific technique for the isolation of individual phenolic compounds, while PLE produced extracts with higher amount of total phenols and higher antioxidant capacity. Both techniques were more effective compared to CE. PLE nettle extract showed antimicrobial activity against bacteria, especially against Gram-negative *Pseudomonas fragi* ATCC 4973 and *Campylobacter jejuni* NCTC 11168 strains. This suggests that PLE is suitable for obtaining a nettle extract with antioxidant and antimicrobial potential, which as such has great potential for use as a value-added ingredient in the food and pharmaceutical industry.

## 1. Introduction

Nettle (*Urtica dioica* L.) is an annual plant belonging to the genus *Urtica*, family Urticaceae, that widespread in Europe, Asia, Africa and North America. The plant has been traditionally used for medicinal purposes for centuries due to its great biological activity and beneficial effects on human health attributed to its antioxidant, antimicrobial, anti-inflammatory, anti-ulcer activity and analgesic properties [1]. These properties can be ascribed to diverse bioactive molecules present in nettle. The leaves are rich in terpenoids, carotenoids, fatty acids, essential amino acids, chlorophylls, vitamins, carbohydrates, sterols, polysaccharides, isolectins and minerals, and are also a vast source of polyphenols [1,2]. Studies have confirmed the correlation between dietary intake of polyphenols, especially phenolic acids, flavonoids and tannins, and their health promoting effects arise from their potential to scavenge reactive species and therefore prevent oxidative stress and inflammation [3,4]. The phenolic composition of nettle is influenced by the variety, genotype, climate, soil, vegetative state, harvest time, storage, and processing, further differing between different parts of the plant [2]. Generally, leaves are considered to be the richest part of the plant for their phenolic content. Zeković et al. (2017) [5] reported that the predominant phenolic compound in nettle leaves is rutin, followed by sinapic acid. Ince et al. (2012) [6] listed gallic, caffeic, chlorogenic and *p*-coumaric acid as well as naringenin and naringin as the main phenolic compounds in nettle extracts, while Repajić et al. (2021) [7] reported cinnamic acids and flavonols as the most abundant phenolic classes in nettle leaves. Apart from its medicinal and dietary use, nettle shows a great potential for application in the food industry as natural preservative [2]. It possesses a significant antimicrobial activity against both gram-positive and gram-negative bacteria including *Bacillus subtilis*, *Lactobacillus plantarum*, *Pseudomonas aeruginosa* and *Escherichia coli* [8].

Considering notable applications of polyphenol-rich nettle extracts in both the food and pharmaceutical industries, it is of utmost importance to select the optimal methodology for their isolation. In the current environment, the extraction technique to be used is expected to provide high extraction yield and to be non-destructive, time-saving and environmentally friendly [9,10]. Therefore, advanced extraction techniques have emerged, such as supercritical fluid extraction (SFE), pressurized liquid extraction (PLE), microwave-assisted extraction (MAE) and ultrasound-assisted extraction (UAE). MAE and PLE have both been applied for various procedures of plant polyphenols extraction, thereby providing shorter extraction times due to increase of the analyte solubility in the extraction media [11,12]. The mechanism of MAE is based on microwave irradiation leading to homogenous heating of both the sample and the solvent, and consequently a cell disruption caused by internal superheating due to dipole rotation and ionic conduction [13]. On the other hand, PLE owes its effectiveness to the application of high pressure by which solvent is maintained in a liquid state at high temperatures, thus increasing the extraction efficiency [14]. Although these advanced techniques have benefits in comparison with conventional extraction procedures, in order to make the most of their potential, one must carefully select the process parameters, since extraction yield depends to a large extent on extraction conditions (temperature, time, microwave power, pressure, etc.), selection of the proper extraction solvent, as well as on the characteristics of the plant material itself.

Literature does not provide any information on the detailed optimization of MAE of nettle leaves polyphenols. Zeković et al. (2017) [5] compared nettle extracts isolated by UAE, MAE and subcritical water extraction and provided the differences in chemical composition and antioxidant and antimicrobial activity of the obtained extracts but used fixed conditions for all the extractions. Therefore, the aim of this research was to evaluate the effect of the extraction solvent and MAE process parameters on the phenolic content of nettle leaves extracts in order to establish the optimal MAE conditions and compare it to previously optimized PLE procedure and conventional heath-reflux extraction (CE) in terms of individual phenolic profile, total phenolic content, and antioxidant activity. Furthermore, the purpose of the study was to evaluate the antimicrobial activity of the most potent nettle extract against the most common food microbial strains.

## 2. Results

### 2.1. Optimization of MAE of Nettle Leaves’ Polyphenols

In order to evaluate the influence of MAE parameters on the isolation of nettle leaves’ polyphenols, a full factorial design was employed, varying the extraction temperature (40, 60 and 80 °C), irradiation time (5 and 10 min) and microwave power (300 and 600 W). The range of parameters considered for this research was selected upon previous studies and literature reports focused on isolation of plant polyphenols [13,15,16,17,18]. Additionally, extractions were carried out with three different solvents, water, 30% aqueous ethanol and 30% aqueous acetone. According to the literature reports [13,19,20], 30% aqueous ethanol and acetone solutions have been considered as effective solvents for both conventional and advanced extraction techniques for the isolation of plant polyphenols and were therefore compared with pure distilled water for their effectiveness in MAE of nettle leaves’ polyphenols. Results of total phenolic content (TPC) determination in obtained nettle leaves extracts are shown in Table 1.

It can be observed that TPC ranged from 359.51 ± 14.58 to 2368.89 ± 30.11 mg GAE/100 g depending on the applied extraction conditions. These results are in accordance with previous reports on nettle leaves phenolic content [6,21,22].

Statistical analysis (Table 2) showed significant influence of extraction solvent and temperature on TPC of nettle leaves during MAE, while microwave power and irradiation time did not show significant effect.

When using 30% acetone as the extraction solvent, the produced extracts contained the highest TPC content, followed by 30% ethanol, while water was the least effective for the isolation of nettle polyphenols. Acetone suitability for extraction of plant polyphenols has been reported previously [23] and can be attributed to its ability to inhibit and/or break polyphenol-protein interactions and therefore prevent the formation of insoluble complex and consequently increase the polyphenolic yield [24]. According to Rezaie et al. (2015) [25] acetone is suitable for the extraction of non-glycosidic polyphenols due to its poor ability to form hydrogen bonds, so its high effectiveness in extraction of nettle leaves polyphenols can be related to the predominance of phenolic acids and non-glycosylated flavonoid derivates in nettle polyphenolic profile [7].

**Figure 1:**
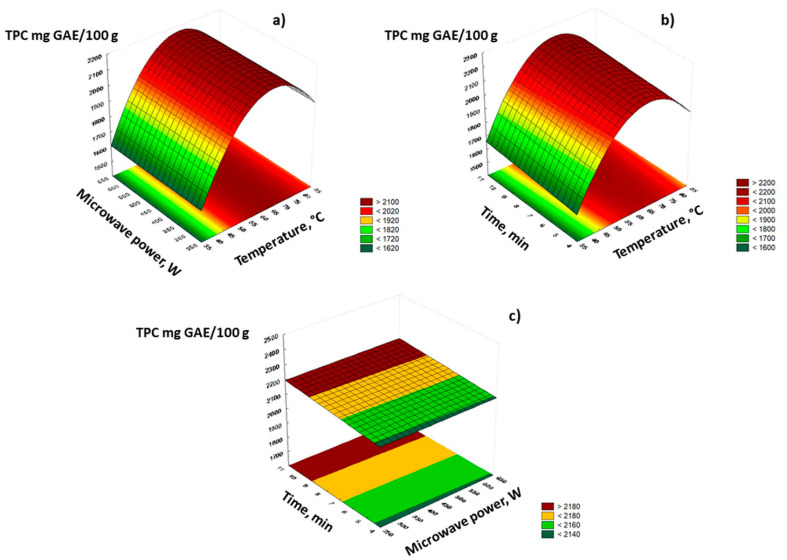
3D graphs of TPC extraction evolution during MAE using 30% acetone as extraction solvent: (**a**) TPC extraction evolution at fixed extraction time of 5 min, (**b**) TPC extraction evolution at fixed microwave power of 300 W, (**c**) TPC extraction evolution at fixed extraction temperature of 60 °C.

Temperature was also shown to be a significant factor for the isolation of nettle leaves polyphenols during MAE. An increase in TPC was observed with temperature elevation from 40 to 60 °C, while a further increase to 80 °C showed no positive effect on total isolated polyphenols (Figure 1). Although higher temperatures generally enhance the extraction efficiency by increasing solute solubility, facilitating molecular motion, providing better mass transfer and reducing solvent viscosity [15,26,27], excessive exposure could also cause degradation and oxidation of polyphenols, so its selection should be based on the characteristics and polyphenolic composition of the subjected plant material. Similar to our conclusions, Alara et al. (2021) [28] found 60 °C to be the optimal temperature for MAE of polyphenols from *Carica papaya* leaves, as a further increase to 70 °C and above caused their degradation. Microwave power and irradiation time did not show significant influence on TPC during MAE (Figure 1). Microwave power is a parameter of MAE that is strongly related to the extraction temperature. The applied power of microwaves directly causes temperature elevation due to the microwave heating of the solvent and plant material. During experiments, temperature was chosen to be the controlled parameter that was kept constant, so selected power was applied only in short increments of time needed to achieve the required temperature. Therefore, according to the statistical analysis, 300 W was selected as optimal for TPC extraction. The same power was applied also for MAE of polyphenols from olive leaves [29]. Obtained results have further shown that there was no significant difference between MAE during 5 and 10 min, indicating 5 min irradiation time as adequate for the isolation of nettle leaves polyphenols. Likewise, Alara et al. (2021) observed the increase in TPC of papaya leaves in single-factor experiments up to 4 min irradiation time at 50 °C and 300 W, followed by reduction in prolonged exposure to microwaves. Relatively short extraction exposure, similar to our observations, has been reported for TPC of *Chromolaena odorata* leaves, namely 3 min at 493 W [30] as well as 5 min at 97 °C for cocoa bean waste [31] and 4 min at 600 W for pomegranate peels [16].

### 2.2. Comparison of Different Extraction Techniques for the Isolation of Nettle Leaves’ Polyphenols

After conducting all MAE trials, the obtained TPC values have shown that optimal MAE conditions for the isolation of nettle leaves polyphenols are 30% acetone as extraction solvent, 60 °C, 300 W microwave power and 5 min irradiation time, resulting in TPC of 2368.89 mg GAE/100 g. In order to provide better insight into impact of advanced extraction techniques on nettle leaves’ polyphenols, extract obtained by MAE at optimal conditions was compared to extracts attained by previously optimized PLE (110 °C, static time 10 min and 4 extraction cycles) and CE (20 min), where all extractions were conducted with 30% acetone as the extraction solvent. These extracts were analyzed for their TPC, individual phenolic profile by UPLC MS^2^ and antioxidant capacity by ORAC (Table 3).

The TPC of nettle leaves extracts was the highest in the one obtained by PLE, followed by MAE, while it was the lowest in one obtained by CE, although with no significant difference between MAE and CE. These results suggest that employment of higher temperatures under high pressure during PLE enhances the solubility of phenolic compounds and desorption kinetics from the plant material [32] in comparison to microwave heating at a moderate temperature of 60 °C. In accordance with our findings, Taamalli et al. (2012) [11] reported higher extraction yield for PLE of phenolic compounds from Tunisian olive leaves in comparison with MAE. Similar results were also reported for TPC of *Stevia rebaudiana* leaves extract obtained by PLE [33].

**Figure 2:**
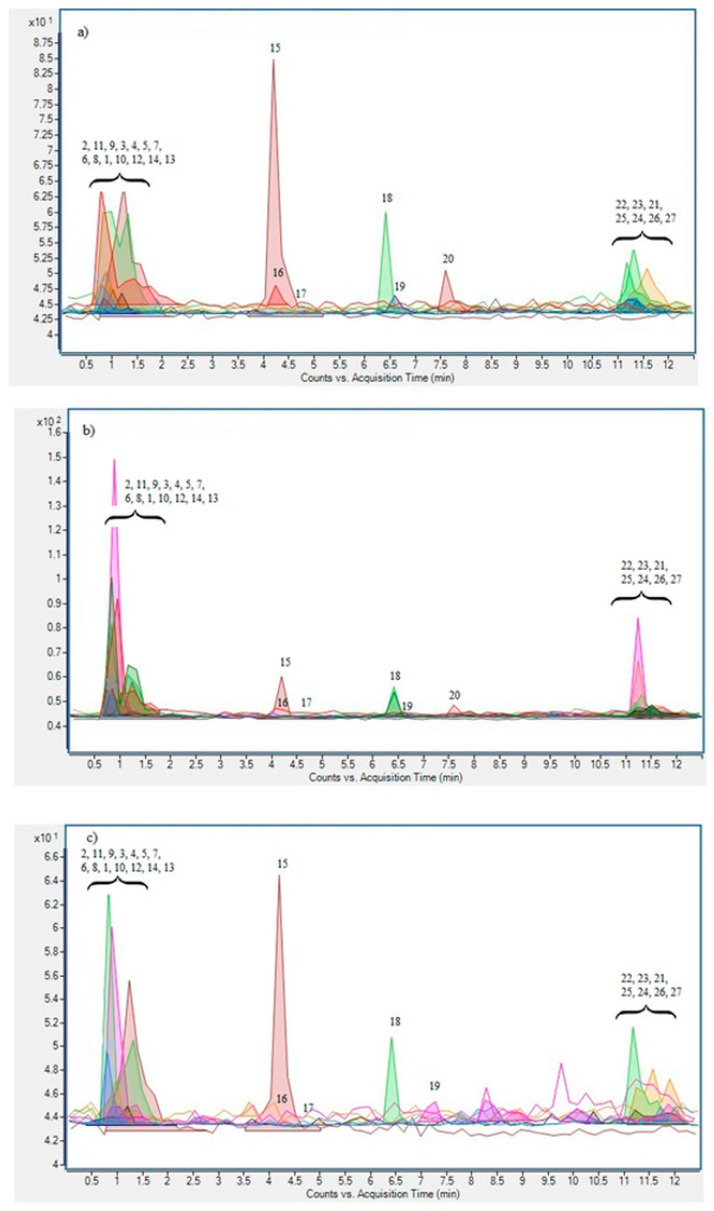
UPLC ESI-MS^2^ chromatogram in MRM acquisition mode from nettle leaves extracts obtained under (**a**) optimized MAE conditions, (**b**) PLE and (**c**) CE. (**1**) Umbelliferone, (**2**) Protocatechuic acid, (**3**) Isorhamnetin 3-*O*-rutinoside, (**4**) Kaempferol-3-*O*-rutinoside, (**5**) Kaempferol hexoside, (**6**) Apigenin hexoside, (**7**) Myricetin, (**8**) Luteolin, (**9**) Caffeic acid, (**10**) Esculetin, (**11**) Gentisic acid, (**12**) Quinic acid, (**13**) Quercetin-3-rutinoside, (**14**) *p*-coumaric acid, (**15**) Cinnamic acid, (**16**) Apigenin, (**17**) Epigallocatechin gallate, (**18**) Ferulic acid, (**19**) Kaempferol pentoside, (**20**) Kaempferol rhamnoside, (**21**) Quercetin-acetyl-rutinoside, (**22**) *p*-hydroxybenzoic acid, (**23**) Gallic acid, (**24**) Epicatechin, (**25**) Kaempferol-pentosyl-hexoside, (**26**) Epicatechin gallate, (**27**) Kaempferol.

The phenolic profile of obtained MAE and PLE extracts did not differ qualitatively, unlike with the CE extract, where some compounds (myricetin and caffeic acid) could not be detected. A detailed description of phenolic composition with accompanying identification pathways for compounds lacking reference standards of nettle leaves from the Croatia region was reported previously [7], and is in accordance with results of this study. A total of 27 phenolic compounds were identified, belonging to the classes of benzoic, cinnamic and other phenolic acids, flavonols, flavan-3-ols, flavones and coumarins (Figure 2). The predominant classes were cinnamic acids represented with cinnamic acid itself as the major compound and flavonols represented by kaempferol aglycone. When observing differences in phenolic profile between different extraction techniques it is interesting to observe that the total sum of identified compounds as well as the concentrations of major phenols such as cinnamic, gallic and ferullic acid, kaempferol-3-rutinoside and kaempferol aglycone were higher in the MAE extract than in the PLE one. A similar trend was reported by Taamalli et al. (2012) [11], showing a larger number of identified phenolic compounds as well as their total concentration in MAE extract of olive leaves when compared to extract obtained by PLE. On the contrary, compounds like gentisic, *p*-hydroxybenzoic and caffeic acid, quercetin-3-rutinoside and epicatechin were higher in PLE extract. Rodriguez-Perez et al. (2016) [12] reported MAE as the more favorable technique for the extraction of kaempferol, quercetin-3-*O*-glucoside and kaempferol-3-*O*-glucoside isomers from *Moringa oleifera* leaves than PLE, confirming our observations regarding the kaempferol and its glycosides. Generally, it is expected that flavonoids are less stabile compounds than phenolic acids due to a larger number of hydroxyl groups and thereforewould be better extracted at lower and moderate temperatures like the ones applied in MAE than in PLE [34]. Indeed, results mostly followed that trend, with the exception of quercetin-3-rutinoside. Therefore, most of the current research could not confirm a universal pattern for the preferred technique regarding the individual phenolic compounds [12,35]. Both advanced techniques were more effective in isolation of all observed polyphenols when compared to CE, with the exception of quinic acid, which was found in significantly higher concentrations in the extract obtained by CE. This is in accordance with previous reports confirming the advantages of non-conventional extraction techniques in terms of producing higher extraction yields and larger number of isolated compounds than conventional ones [36,37,38].

Furthermore, our results indicated an interesting instance. A discrepancy was observed between the amount of TPC and sum of individual phenolic compounds as well as within the efficiency of tested techniques that arises from these results. All extracts showed very high TPC determined spectrophotometrically with the Folin-Ciocalteu method, but a significantly lower sum of the total identified phenolic compounds. It is known that the Folin-Ciocalteu reagent is not as specific as chromatographic techniques, but these differences in nettle extracts were especially evident. As nettle leaves are a rich source of plant pigments (chlorophylls), it is possible that chlorophylls reacted with the Folin-Cioacalteu reagent and apparently increased TPC values. This was also reported previously by El-Hamidi et al. (2016) [39]. Additionally, the interaction of chlorophyll pigments could explain why PLE produced extracts with higher TPC than MAE, although with lower content of individual polyphenols, as PLE has been reported to be an effective method for the isolation of chlorophylls [40,41,42]. Moreover, the Folin-Ciocalteu method has been reported to have disadvantages in terms of interfering with compounds such as sugars, organic acids, ascorbic acid, aromatic amines and Fe(II), which can also be the cause of the high TPC values in nettle leaves extracts [43].

The antioxidant capacity of three different nettle leaf extracts was evaluated using an ORAC assay, as previous studies have confirmed that this assay is biologically most relevant and can measure both lipophilic and hydrophilic antioxidants. It is one of the hydrogen atom transfer assays relevant to the ability of antioxidants to break radical chains, as the peroxyl radical it measures is the predominant free radical in lipid oxidation in foods and biological systems [43,44].The results for ORAC, shown in Table 3, followed the same pattern as their TPC, with PLE extract having significantly higher ORAC value than MAE and CE. This implicates the involvement of different bioactive compounds in total antioxidant capacity of the extract, not exclusively polyphenols, as it was evident that the PLE technique isolated more non-phenolic compounds than MAE and CE. Therefore, when selecting the technique for the production of nettle extract with high biological activity, one should consider the employment of PLE.

### 2.3. Antimicrobial Activity of Nettle Leaves Extract

As PLE extract of nettle leaves showed to be a rich source of bioactive compounds with high antioxidant capacity, it was analyzed for its antimicrobial activity in order to provide information on its possible usage as a natural antimicrobial agent. Antimicrobial activity was determined against gram-positive and gram-negative bacterial strains, as well as against two yeast strains, which makes this important for the food industry as foodborne pathogens or food spoilage microorganisms. The results are presented in Table 4.

PLE extract showed antimicrobial activity against some of the selected bacteria. The highest antimicrobial activity was observed against Gram-negative *P. fragi* and *C. jejuni* strains with MIC less than 0.512 mg/mL indicating significant antimicrobial activity [45]. Gram-negative *Shewanella* strains and Gram-positive *S. aureus* were also sensitive to the extract, which showed moderate antimicrobial activity. For these bacteria the extract had bactericidal activity in addition to bacteriostatic activity. No inhibitory effect was observed against *L. innocua*, *E. coli* and against both yeast strains, *C. albicans* and *P. anomala*. The antimicrobial activity of nettle extracts has been previously observed by several researchers. Similar MICs for nettle extract to ours were determined by Sterniša et al. (2020) [46] (0.39–0.78 mg/mL). They also determined higher antimicrobial activity of nettle extract when compared to oregano extract. On the other hand, the MICs of nettle leaves extract were higher (6.25–50 mg/mL) in the research of Rajput et al. (2019) [47] where *E. coli* was the most sensitive among strains tested. Mahmoudi et al. (2014) [48] reported that aqueous and ethanolic nettle extracts had an inhibitory effect against both Gram-positive and Gram-negative bacteria, as well as against *C. albicans*. Gram-positive bacteria (*S. aureus* and *L. monocytogenes*) were more sensitive to extracts from nettle leaves, while Gram-negative bacteria (*P. vulgaris* and *K. pneumoniae*) to extracts from nettle roots. Zeković et al. (2017) [5] observed an impact of non-conventional extraction techniques on antimicrobial activity of nettle leaves extracts. Their results revealed high bioactive potential of all obtained extracts, among which subcritical water extraction provided the most potent extract, especially against *S. aureus*.

As standard active compounds, we tested kaempferol and cinnamic acid, which have known antimicrobial activity [49,50] and which were also the two main components of the extract. The analysis was carried out on selected gram-positive and gram-negative bacteria, *S. aureus* and *P. fragi*, respectively. None of the pure active compounds reached the MIC of the extract. The MIC for cinnamic acid was 5 mg/mL for both bacteria and also for kaempferol for *P. fragi*. For *S. aureus*, the MIC of kaempferol was not determined (> 5 mg/mL). Cinnamic acid and kaempferol were by far the most abundant bioactive compounds found in nettle leaves extract; both are known for their beneficial effects [49,51,52]. These two bioactive compounds are thus most likely not solely responsible for the antimicrobial activity of the extracts, although they are abundant. Therefore, the antimicrobial activity of the PLE nettle extract is probably the result of synergistic activity of all present bioactives, as reported previously [53]. The nettle extract was particularly active against *P. fragi* and *C. jejuni*. *P. fragi* is an important cause of spoilage of proteinaceous foods in the food industry, so the activity of the nettle leaves extract against it is of particular interest due to the generally higher resistance of this Gram-negative bacteria to antimicrobial agents. Some researchers [46,54] attribute this phenomenon to the high enzymatic activity of some microorganisms, including the genus *Pseudomonas*. It is assumed that the microbial enzymes convert the form of glycosylated phenolic compounds into an aglycone form, thereby releasing phenolics with high antimicrobial activity and increasing their bioactivity.

## 3. Materials and Methods

### 3.1. Plant Material

Wild nettle leaves (*Urtica dioica* L.) were collected in July 2019 in the Lika area of Croatia. Plant material was identified using the usual keys and iconographies outlined by the Department of Vegetable Crops, Faculty of Agriculture, University of Zagreb (Croatia). Immediately after harvesting, the nettle leaves were separated from stalks and freeze-dried (Alpha 1–4 LSCPlus, Martin Christ Gefriertrocknungsanlagen GmbH, Osterode am Harz, Germany) for 24 h until final moisture content of 3%. Afterwards, dry leaves were ground using an electric mill and stored in a dry and dark place until analyzed.

### 3.2. Chemicals and Reagents

Ethanol and acetone used for the extractions and analysis were HPLC grade, purchased from Gram-mol Ltd. (Zagreb, Croatia). Acetonitrile (HPLC grade) was purchased from J.T. Baker Chemicals (Deventer, Netherlands) and formic acid (98–100%) from T.T.T. Ltd. (Sveta Nedjelja, Croatia). Distilled water was of Milli-Q quality (Millipore Corp., Bedford, NY, USA). Anhydrous sodium carbonate (99.5%) and sodium phosphate (96%) were obtained from Kemika (Zagreb, Croatia), sodium chloride and Folin-Ciocalteu reagent from Merck (Darmstadt, Germany), fluorescein sodium salt from Honeywell Riedel-de-Haën (Bucharest, Romania), Trolox (6-hydroxy-2,5,7,8-tetramethylchroman-2-carboxylic acid) from Acros Organics (Thermo Fisher Scientific, Geel, Belgium) and 2,20-Azobis (2-amidinopropane) hydrochloride from Sigma-Aldrich (Steinheim, Germany). Dimethyl sulfoxyde (DMSO) was obtained from Merck and iodonitrotetrazolium chloride (95%) from Sigma Aldrich. 2-*p*-iodophenyl-3-*p*-nitrophenyl-5-phenyl tetrazolium chloride and resazurin were from Sigma Aldrich (St. Louis, MO, USA).

Tryptic Soy Agar, Tryptic Soy Broth and Karmali were purchased from Biolife (Milan, Italy), Mueller-Hinton Agar from bioMérieux (Marcy-l′Étoile, France), Mueller-Hinton Broth from Oxoid (Hampshire, UK), Malt Extract Agar and Malt Extract Broth from Merck (Kenilworth, NJ, USA).

Commercial phenolic compounds’ standards of gallic, cinnamic, caffeic, *p*-coumaric, ferullic and quinic acid, kaempferol-3-rutinoside, myricetin and quercetin-3-glucoside were purchased from Sigma-Aldrich, epicatechin, epigallocatechingallate, epicatechingallate, luteolin, apigenin and esculetin from Extrasynthese (Genay, France), quercetin-3-rutinoside from Acros Organics and kaempferol from Cayman Chemicals (Ann Arbor, MI, USA).

### 3.3. Extraction Procedures

#### 3.3.1. MAE

Polyphenols from wild nettle leaves were extracted using the microwave reactor Ethos Easy (Milestone, Sorisole, Italy) with adjustable microwave power up to 1900 W, operating at 2450 MHz. General extraction parameters were kept constant: time required for temperature achievement—2 min, ventilation after the extraction—1 min and stirring 50%. Extractions were performed according to the experimental design shown in Table 1, varying the extraction temperature (40, 60 and 80 °C), irradiation time (5 and 10 min) and microwave power (300 and 600 W) with three different extraction solvents (water, 30% ethanol and 30% acetone). During all trials the temperature was kept as a constant parameter.

Approximately 1 g of sample was mixed with 40 mL of extraction solvent, placed in extraction cell with magnetic stirrer and positioned on the rotor of microwave reactor for extraction process. Afterwards, extracts were filtered through Whatman No. 40 filter paper (Whatman International Ltd., Kent, UK) into 50 mL volumetric flasks, made up to volume with solvent, transferred to plastic Falcon tubes and stored at −18 °C in a nitrogen gas atmosphere until analyzed.

#### 3.3.2. PLE

For comparison of MAE efficiency in isolation of wild nettle leaves’ polyphenols, PLE was also performed with three extraction solvents (water, 30% ethanol and 30% acetone) using Dionex™ ASE™ 350 Accelerated Solvent Extractor (Thermo Fisher Scientific Inc., Sunnyvale, CA, USA) in 34 mL stainless steel cells. Cells were fitted with two cellulose filters (Dionex™ 350/150 Extraction Cell Filters, Thermo Fisher Scientific Inc., Waltham, MA, USA) and filled with 1 g of sample mixed with 2 g of diatomaceous earth. Extractions were performed at previously established optimized conditions by Repajić et al. (2020) [55], namely temperature 110 °C, static time 10 min and 4 extraction cycles, pressure 10.34 MPa, 30 s of purge with nitrogen and 50% volume flush. Extracts were collected in 250 mL glass vials, transferred to 50 mL volumetric flasks, made up to volume with solvent and stored at −18 °C in a nitrogen gas atmosphere until analyzed.

#### 3.3.3. CE

For comparison with advanced extraction techniques, wild nettle leaves’ polyphenols were further extracted with the same three extraction solvents using the heat-reflux method. Approximately 1 g of the sample was mixed with 40 mL of extraction solvent in a round bottom Erlenmeyer flask. The mixture was extracted for 20 min with reflux, filtered through filter paper, made up to 50 mL with the appropriate extraction solvent and stored at −18 °C in a nitrogen gas atmosphere until analyzed.

### 3.4. Polyphenols’ Extracts Analysis

#### 3.4.1. Determination of TPC

The TPC of wild nettle leaves extracts was determined by the spectrophotometric Folin-Ciocalteu method described by Shortle et al. (2014) [56], with some modifications. The aliquot of 100 µL of each extract was mixed with 200 µL of Folin-Ciocalteu reagent and 2 mL of distilled water. After 3 min, 1 mL of 20% sodium carbonate solution was added. Mixtures were thermostated at 50 °C for 25 min in water bath and afterwards absorbance was measured at 765 nm. A blank was prepared with the same reagents using the appropriate extraction solvent volume instead of extract. TPC was expressed as mg gallic acid equivalents (GAE) per 100 g of sample according to the gallic acid calibration curve.

#### 3.4.2. UPLC/ESI MS^2^ Analysis

The identification and quantification of polyphenols in optimized MAE, PLE and CE extracts of wild nettle leaves was performed by UPLC/ESI-MS^2^ analysis on an Agilent series 1290 RRLC instrument (Agilent, Santa Clara, CA, USA) connected to a triple quadrupole mass spectrometer (6430) with ESI ion source. Ionization was done by ESI in both positive and negative mode (*m*/*z* 100 to 1000) with nitrogen (99.999%, Messer, Croatia) as inducing cone and collision gas under following conditions: positive/negative capillary voltage, 4000/3500 V; drying gas temperature of 300 °C with a flow rate of 11 L/h and nebulizer pressure 40 psi. Chromatographic separations were performed on a Zorbax Eclipse Plus C18 column (Agilent, 100 mm × 2.1 mm; 1.8 µm particle size) at 35 °C with 2.5 µL injection volume. Data were acquired and processed using Agilent MassHunter Workstation Software (ver. B.04.01, Agilent, Santa Clara, CA, USA). The solvent composition and methodology used was described previously by Elez Garofulić et al. (2018) [57]. Identification and quantitative determination were carried out using the calibration curves of the standards: gallic, caffeic, *p*-coumaric and ferullic acid, kaempferol-3-rutinoside, myricetin, quercetin-3-glucoside, quercetin-3-rutinoside, epicatechin, epigallocatechingallate, epicatechingallate, luteolin, apigenin and esculetin. Quality parameters for the analytical method including calibration curves, instrumental detection (LOD) and quantification (LOQ) limits were reported previously [57]. For compounds lacking reference standards identification was performed according to the mass spectral data and literature reports on mass fragmentation patterns, while quantification was done as follows: protocatechuic, gentisic and *p*-hydroxybenzoic acid according to the gallic acid calibration curve, isorhamnetin rutinoside and quercetin acetylrutinoside according to the quercetin-3-glucoside, kaempferol hexoside, kaempferol pentoside, kaempferol rhamnoside, kaempferol pentosylhexoside and kaempferol according to the keampferol-3-rutinoside, apigenin hexoside according to the apigenin and umbelliferone according to the esculetin calibration curve. All results were expressed as mg per 100 g of sample.

#### 3.4.3. ORAC Assay

The antioxidant capacity of the extracts was assessed by the oxygen radical absorbance capacity (ORAC) assay based on a previously reported method [44,58] with minor modifications. For the analysis, an automated plate reader (BMG LABTECH, Offenburg, Germany) with 96-well plates was used and data were analyzed by MARS 2.0 software (BMG LABTECH, Offenburg, Germany). Diluted samples were added in a black plate containing a fluorescein solution (70.3 nM) and incubated for 30 min at 37 °C. After the first three cycles (representing the baseline signal), 240 mM AAPH was injected into each well to initiate the peroxyl radical generation. Different dilutions of Trolox (3.12–103.99 µM) were used on each plate as a reference standard. Fluorescence intensity (excitation at 485 nm and emission at 528 nm) was monitored every 90 s over a total measurement period of 120 min and the results were expressed as µmol of Trolox equivalents (TE)/100 g.

#### 3.4.4. Antimicrobial Activity

Microbiological analysis was performed in extract containing the highest TPC content (PLE extract). Dried extract (SAVANT SPD 2010 SpeedVac Concentrator, Thermo Fisher Scientific Inc., Sunnyvale, CA, USA) and pure compounds were dissolved in DMSO to prepare stock solutions.

Selected food spoilage microbial strains or foodborne pathogens (*Listeria innocua* ŽM39, *Staphylococcus aureus* ATCC 25,923, *Escherichia coli* ATCC 11,229, *Pseudomonas fragi* ATCC 4973, *Shewanella putrefaciens* ŽM654, *Shewanella xiamenensis* ŽM655, *Shewanella baltica* NCTC 10,735, *Campylobacter jejuni* NCTC 11,168, *Candida albicans* ZIM 2202 and *Pichia anomala* ZIM 1769) were included in the experiment. Microorganisms were obtained from the collection of microorganisms of the Laboratory for Food Microbiology at the Department of Food Science, Biotechnical Faculty, University of Ljubljana, Slovenia (designation ŽM), the American Type Culture Collection (designation ATCC), the National Collection of Type Cultures (designation NCTC) and the Slovenian Collection of Industrial Microorganisms (designation ZIM). Tryptic Soy Agar, Karmali, Mueller-Hinton Agar and Malt Extract Agar were used for revitalization of frozen strains. Strains were grown under following conditions: *L. innocua*, *S. aureus* and *E. coli* 24 h at 37 °C, *P. fragi*, *S. putrefaciens*, *S. xiamenensis* and *S. baltica* 24 h at 30 °C, *C. jejuni* 24 h at 42 °C under microaerophilic conditions (5% O_2_, 10% CO_2_, 85% N_2_), *C. albicans* and *P. anomala* 48 h at 30 °C. After incubation, the culture was transferred into 0.9% NaCl solution to obtain optical density (OD) at 600 nm 0.090–0.110 (for bacterial strains) or 0.170–0.200 (for yeast strains). The suspension was 100-fold diluted in broth media to obtain an inoculum concentration of approximately 10^5^ CFU/mL. The plate count method was used to determine the exact concentration of microbial cells in inocula.

Minimal inhibitory concentrations (MICs) of the extract and pure compounds were determined using the broth microdilution method as previously described by Klančnik et al. (2010) [59]. Twofold dilutions of the extract or pure compound were done in sterile 96-well microtiter plates with final volume of 50 μL. Then the same volume of inoculum was added into each well. Positive control contained 50 μL of broth media and 50 μL of inoculum. Negative control contained 100 μL of broth media. The microtiter plates were mixed on a microtiter plate shaker (600 rpm, 1 min) (Eppendorf Thermomixer Comfort, Hamburg, Germany) and incubated for 24 h. MICs of the extract/pure compounds were the lowest concentrations at which no microbial growth was observed after adding 2-p-iodophenyl-3-p-nitrophenyl-5-phenyl tetrazolium chloride or resazurin as indicators. The interpretation of obtained MIC values was based on Tamokou et al. (2017) [45], according to which the extract is very active at MIC < 0.1 mg/mL, significantly active at 0.1 mg/mL ≤ MIC ≤ 0.512 mg/mL, moderately active at 0.512 mg/mL < MIC ≤ 2.048 mg/mL, not very active at MIC > 2.048 mg/mL and not active at MIC > 10 mg/mL. Minimal bactericidal or fungicidal concentration (MBC/MFC) of the extract/pure compound was the lowest concentration at which no microbial growth on agar after sub-cultivation of bacterial suspension was observed.

### 3.5. Statistical Analysis

Statistical analysis was carried out using Statistica ver. 10.0 software (Statsoft Inc., Tulsa, OK, USA). All extractions and all analysis were performed in duplicate. A mixed 2- and 3-level full factorial design comprising 36 experimental trials was employed for evaluation of the effect of four independent variables (solvent, temperature, irradiation time and microwave power) on the TPC of nettle leaves during MAE (Table 1). Descriptive statistics was used to assess the basic information about the experimental data set, whereas differences in applied MAE treatments were tested by multifactorial analysis of variance (MANOVA) and post hoc Tukey’s honestly significant difference (HSD) test. In order to compare effectiveness of different extraction techniques, means were compared using one-way analysis of variance (ANOVA) and post hoc Tukey’s HSD test. All differences were considered significant at a level of *p* ≤ 0.05.

## 4. Conclusions

MAE was optimized for the isolation of nettle leaves’ polyphenols, producing the highest extraction yields at 60 °C, 300 W and 5 min with 30% aqueous acetone as the extraction solvent. In comparison with CE, optimized MAE and PLE as advanced extraction techniques showed to be fast and effective methods resulting in extracts with higher phenolic content and with higher antioxidant capacity. The optimized MAE procedure showed to be more specific towards the isolation of individual polyphenols, while PLE isolated higher amounts of total phenols and produced extracts with slightly higher antioxidant capacity, thereby implicating the predominance of PLE for the isolation of total bioactive compounds present in nettle. PLE extract showed antimicrobial activity against *P. fragi*, *C. jejuni*, *S. aureus* and *Shewanella* strains. These results show that nettle leaves extract could be used as a potent agent for prolonging the shelf life of foods and reducing foodborne infections. However, as this study suggested, both the antioxidant and antimicrobial activity of nettle extract are a result of involvement of different bioactive compounds and therefore future studies should be employed on the effect of advanced extraction techniques on other bioactives present in nettle other than polyphenols.

## Figures and Tables

**Table 1:** Total phenolic content (TPC) in nettle leaves extracts obtained under different conditions of MAE according to the full factorial design.

Solvent	Temperature, °C	Microwave Power, W	Time, min	TPC, mg GAE/100 g
Water	40	300	5	490.12 ± 9.47
			10	530.17 ± 22.84
		600	5	359.51 ± 14.58
			10	398.48 ± 1.96
	60	300	5	1527.78 ± 28.06
			10	1489.61 ± 25.21
		600	5	1274.32 ± 23.01
			10	1161.25 ± 24.60
	80	300	5	1243.47 ± 8.05
			10	1598.67 ± 38.39
		600	5	1476.47 ± 2.95
			10	980.27 ± 13.22
30% Ethanol	40	300	5	1623.83 ± 13.81
			10	1714.06 ± 66.06
		600	5	1789.61 ± 34.07
			10	1765.06 ± 29.46
	60	300	5	1705.78 ± 65.31
			10	1698.21 ± 28.11
		600	5	1670.68 ± 16.02
			10	1954.55 ± 53.50
	80	300	5	1584.62 ± 38.12
			10	1461.67 ± 18.08
		600	5	1893.07 ± 72.25
			10	1837.77 ± 54.96
30% Acetone	40	300	5	1565.56 ± 4.01
			10	2007.04 ± 42.77
		600	5	1820.70 ± 42.41
			10	1784.35 ± 44.34
	60	300	5	2368.89 ± 30.11
			10	2024.00 ± 38.11
		600	5	2063.77 ± 11.96
			10	2211.71 ± 19.97
	80	300	5	2065.42 ± 37.93
			10	2040.21 ± 16.07
		600	5	2063.25 ± 33.40
			10	2135.76 ± 35.77

Results are expressed as mean ± standard deviation.

**Table 2:** Total phenolic content (TPC) in nettle leaves extracts influenced by the main parameters of MAE.

MAE Parameters	TPC, mg GAE/100 g
Solvent	*p* < 0.05 *
Water	1044.18 ± 6.98 ^a^
30% ethanol	1724.91 ± 6.98 ^b^
30% acetone	2012.55 ± 6.98 ^c^
Temperature, °C	*p* < 0.05 *
40	1320.71 ± 6.98 ^a^
60	1762.55 ± 6.98 ^c^
80	1698.39 ± 6.98 ^b^
Microwave power, W	*p* = 0.50
300	1596.62 ± 5.70 ^a^
600	1591.14 ± 5.70 ^a^
Time, min	*p* = 0.16
5	1588.16 ± 5.70 ^a^
10	1599.60 ± 5.70 ^a^

* Statistically significant variable at *p* ≤ 0.05. Results are expressed as mean ± standard error. Means with the same letter within the column are not significantly different at *p* ≤ 0.05 by Tukey’s HSD test.

**Table 3:** Polyphenolic profile including mass spectrometric data and concentration of identified individual compounds, total phenolic content (TPC) and ORAC values of nettle leaves extracts obtained under optimized MAE conditions, PLE and CE.

	Mass Spectrometric Data		Concentration (mg/100 g)		
	Percursor Ion *(m/z*)	Fragment Ion (*m/z*)	MAE	PLE	CE
Benzoic acids					
Protocatechuic acid	153	109	4.90 ± 0.04 ^c^	4.27 ± 0.08 ^b^	2.25 ± 0.04 ^a^
Gentisic acid	153	109	2.10 ± 0.06 ^a^	8.12 ± 0.20 ^b^	2.17 ± 0.03 ^a^
*p*-hydroxybenzoic acid	137	93	11.88 ± 0.59 ^a^	16.12 ± 0.51 ^b^	10.42 ± 0.42 ^a^
Gallic acid *	169	125	9.76 ± 0.44 ^c^	3.24 ± 0.08 ^a^	5.81 ± 0.14 ^b^
Cinnamic acids					
Caffeic acid *	179	135	1.25 ± 0.04 ^a^	5.97 ± 0.34 ^b^	nd
*p*-coumaric acid *	163	119	7.04 ± 0.35 ^b^	8.07 ± 0.30 ^b^	1.78 ± 0.04 ^a^
Cinnamic acid *	147	103	178.29 ± 4.85 ^b^	121.50 ± 5.01 ^a^	148.52 ± 9.40 ^a^
Ferullic acid *	193	178	15.22 ± 0.41 ^c^	4.74 ± 0.21 ^a^	7.28 ± 0.38 ^b^
Other phenolic acids					
Quinic acid *	191	85	1.59 ± 0.04 ^a^	4.02 ± 0.13 ^b^	10.71 ± 0.61 ^c^
Flavonols					
Isorhamnetin 3-*O*-rutinoside	625	317	1.65 ± 0.06 ^a^	2.63 ± 0.10 ^b^	2.69 ± 0.11 ^b^
Kaempferol-3-*O*-rutinoside *	595	287	10.56 ± 0.55 ^c^	4.94 ± 0.08 ^b^	3.43 ± 0.06 ^a^
Kaempferol hexoside	449	287	3.61 ± 0.13 ^b^	5.29 ± 0.24 ^c^	2.72 ± 0.04 ^a^
Myricetin *	319	273	2.59 ± 0.03 ^a^	4.21 ± 0.07 ^b^	nd
Quercetin-3-rutinoside *	611	303	18.68 ± 1.02 ^a^	42.38 ± 1.74 ^b^	17.56 ± 0.68 ^a^
Kaempferol pentoside	419	287	2.49 ± 0.04 ^c^	2.34 ± 0.04 ^b^	0.46 ± 0.01 ^a^
Kaempferol rhamnoside	433	287	4.48 ± 0.17 ^b^	4.22 ± 0.10 ^b^	1.33 ± 0.04 ^a^
Quercetin-acetyl-rutinoside	653	303	12.14 ± 0.95 ^a^	11.19 ± 0.55 ^a^	10.43 ± 0.64 ^a^
Kaempferol-pentosyl-hexoside	597	303	3.79 ± 0.24 ^b^	3.09 ± 0.08 ^a^	3.27 ± 0.08 ^ab^
Kaempferol	285	285	116.13 ± 7.69 ^a^	107.89 ± 4.91 ^a^	94.03 ± 5.81 ^a^
Flavan-3-ols					
Epigallocatechingallate *	459	289, 139	4.16 ± 0.06 ^c^	1.76 ± 0.06 ^a^	2.89 ± 0.10 ^b^
Epicatechin *	291	139	4.45 ± 0.11 ^a^	30.77 ± 1.71 ^b^	3.34 ± 0.07 ^a^
Epicatechingallate *	443	291	2.18 ± 0.08 ^c^	1.76 ± 0.03 ^b^	1.11 ± 0.03 ^a^
Flavones					
Apigenin hexoside	433	271	2.25 ± 0.08 ^b^	4.39 ± 0.04 ^c^	1.75 ± 0.06 ^a^
Luteolin *	287	153	5.40 ± 0.16 ^c^	0.64 ± 0.01 ^a^	1.93 ± 0.04 ^b^
Apigenin *	271	153	14.07 ± 0.30 ^b^	15.31 ± 0.82 ^b^	3.58 ± 0.10 ^a^
Coumarins					
Umbelliferone	161	133	2.18 ± 0.03 ^b^	3.36 ± 0.07 ^c^	1.83 ± 0.04 ^a^
Esculetin *	177	133	2.91 ± 0.08 ^b^	2.91 ± 0.03 ^b^	1.71 ± 0.04 ^a^
Total UPLC MS^2^ identified compounds (mg/100 g)			445.75 ± 8.65 ^b^	425.13 ± 16.87 ^b^	343.00 ± 19.01 ^a^
TPC (mg GAE/100 g)			2368.89 ± 30.11 ^a^	3301.20 ± 171.16 ^b^	2082 ± 84.38 ^a^
ORAC (µmol TE/100 g)			929.80 ± 6.28 ^a^	1074.40 ± 31.20 ^b^	925.60 ± 6.70 ^a^

* identification confirmed using authentic standards; nd—not detected. Results are expressed as mean ± standard deviation. Means with the same letter within the row are not significantly different at *p* ≤ 0.05 by Tukey’s HSD test.

**Table 4:** Antimicrobial activity of nettle leaves PLE extract described as MIC and MBC/MFC values [mg/mL].

Microbial Strain	Nettle Leaves PLE Extract	
	MIC [mg/mL]	MBC/MFC [mg/mL]
*Staphylococcus aureus* ATCC 25923	2	2
*Listeria innocua* ŽM39	nd	nd
*Escherichia coli* ATCC 11229	nd	nd
*Pseudomonas fragi* ATCC 4973	0.5	1
*Shewanella putrefaciens* ŽM654	2	2
*Shewanella xiamenensis* ŽM655	2	4
*Shewanella baltica* NCTC 10735	4	nd
*Campylobacter jejuni* NCTC 11168	0.5	1
*Candida albicans* ZIM 2202	nd	nd
*Pichia anomala* ZIM 1769	nd	nd

nd—not determined, >4 mg/mL.

## Data Availability

Data is contained within the article.

## Sample Availability

Samples of the compounds are not available from the authors.
